# Supplementary material for: What do we really know about the appropriateness of radiation emitting imaging for low back pain in primary and emergency care? A systematic review and meta-analysis of medical record reviews
Source: PLoS One. 2019 Dec 5;14(12):e0225414. doi: 10.1371/journal.pone.0225414 (PMC6894771; doi:10.1371/journal.pone.0225414)
Supplement: S1 Appendix — (DOCX) [file pone.0225414.s001.docx]

**Search Strategies**

Pubmed

("Back Pain"[Mesh] OR "back pain"[tiab] OR backache[tiab] OR "back pains"[tiab] OR backaches[tiab] OR "back aches"[tiab] OR dorsalgia[tiab]) AND ("Guidelines as Topic"[Mesh] OR "Practice Guideline"[Publication Type] OR advice[tiab] OR treatment[tiab] OR options[tiab] OR policy[tiab] OR protocol[tiab] OR Guidelines[tiab] OR "decision tool"[tiab] OR "decision aid"[tiab] OR algorithm[tiab]) AND ("Guideline Adherence"[Mesh] OR "guideline adherence"[tiab] OR "policy compliance"[tiab] OR "protocol compliance"[tiab] OR "protocol adherence"[tiab] OR "Institutional adherence"[tiab] OR "Institutional compliance"[tiab] OR comply[tiab] OR compliant[tiab] OR conform[tiab] OR conformance[tiab] OR appropriateness[tiab] OR justif*[tiab])

Embase

(('backache'/exp OR 'backache'/de OR backache*:ti,ab OR 'back'/exp OR back) AND pain*:ti,ab OR 'back pain syndrome':ti,ab OR backpain*:ti,ab OR dorsalgia*:ti,ab OR 'pain, back':ti,ab) AND (('clinical practice guidelines':ti,ab OR guideline*:ti,ab OR 'guidelines as topic':ti,ab OR 'practice'/exp OR practice) AND guideline*:ti,ab OR 'practice guidelines as topic':ti,ab) AND (adherence*:ti,ab OR compliance*:ti,ab OR conform*:ti,ab OR justif*:ti,ab)

CINAHL

(MH "Back Pain+" OR TI "back pain*" OR AB "back pain*" OR TI backache* OR AB backache* OR TI "back ache*" OR AB "back ache*" OR TI dorsalgia OR AB dorsalgia) AND (MH "Practice Guidelines" OR PT "Practice Guideline" OR TI advice OR AB advice OR TI treatment OR AB treatment OR TI options OR AB options OR TI policy OR AB policy OR TI protocol OR AB protocol OR TI guidelines OR AB guidelines OR TI "decision tool" OR AB "decision tool" OR TI "decision aid" OR AB "decision aid" OR TI algorithm OR AB algorithm) AND (MH "Guideline Adherence" OR TI "guideline adherence" OR AB "guideline adherence" OR TI "policy compliance" OR AB "policy compliance" OR TI "protocol compliance" OR AB "protocol compliance" OR TI "protocol adherence" OR AB "protocol adherence" OR TI "institutional adherence" OR AB "institutional adherence" OR TI "institutional compliance" OR AB "institutional compliance" OR TI comply OR AB comply OR TI compliant OR AB compliant OR TI conform OR AB conform OR TI conformance OR AB conformance OR TI appropriateness OR AB appropriateness OR TI justif* OR AB justif*)
