# Supplementary material for: What do we really know about the appropriateness of radiation emitting imaging for low back pain in primary and emergency care? A systematic review and meta-analysis of medical record reviews
Source: PLoS One. 2019 Dec 5;14(12):e0225414. doi: 10.1371/journal.pone.0225414 (PMC6894771; doi:10.1371/journal.pone.0225414)
Supplement: S2 Appendix — (DOCX) [file pone.0225414.s002.docx]

**Appendix 2.** Studies identified in search strategy (including forward and backward tracking) and the reason(s) they were excluded from descriptive synthesis and meta-analysis.

| **Study** | **Reason for Exclusion** |
| --- | --- |
| **Fullen 2007** | Prospective data collection from GPs, outcome reporting and patient population |
| **Raja 2018** | Aggregate number which included MRI |
| **Charlesworth 2016** | Aggregate number with MRI |
| **Foo 2017** | Aggregate number with MRI |
| **Kost 2015** | Aggregate number with MRI |
| **Lin 2016** | Aggregate number with MRI |
| **Rao 2015** | Aggregate number with MRI |
| **Rego 2016** | Aggregate number with MRI |
| **Bishop 2003** | Guideline year |
| **Buller-Close 2003** | Guideline year |
| **Day 1995** | Guideline year |
| **Deyo 1986** | Guideline year |
| **Eccles 2001** | Guideline year |
| **Espeland 1999** | Guideline year |
| **Espeland 2001** | Guideline year |
| **Gonzalez- Urzelai 2003** | Guideline year, data collection method |
| **Halpin 1991** | Guideline year |
| **Hourcade 2002** | Guideline year |
| **Richards 2002** | Guideline year |
| **Schectman 2003** | Guideline year |
| **Schroth 1992** | Guideline year |
| **Suarez-Almazor 1997** | Guideline year |
| **Tacci 1999** | Guideline year |
