# Supplementary material for: What do we really know about the appropriateness of radiation emitting imaging for low back pain in primary and emergency care? A systematic review and meta-analysis of medical record reviews
Source: PLoS One. 2019 Dec 5;14(12):e0225414. doi: 10.1371/journal.pone.0225414 (PMC6894771; doi:10.1371/journal.pone.0225414)
Supplement: S3 Appendix — (DOCX) [file pone.0225414.s003.docx]

Appendix 3 RECORD and STROBE Checklist Items for Included studies in descriptive synthesis

| Author | Item^x^ | | | | | | | | | | | | | | | | | | | | | | | | | | | | | | | | | | | |
| --- | --- | --- | --- | --- | --- | --- | --- | --- | --- | --- | --- | --- | --- | --- | --- | --- | --- | --- | --- | --- | --- | --- | --- | --- | --- | --- | --- | --- | --- | --- | --- | --- | --- | --- | --- | --- |
|  | 1 | 1.1 | 1.2 | 1.3 | 2 | 3 | 4 | 5 | 6 | 6.1 | 6.2 | 6.3 | 7 | 7.1 | 8 | 9 | 10 | 11 | 12 | 12.1 | 12.2 | 12.3 | 13 | 13.1 | 14 | 15 | 16 | 17 | 18 | 19 | 19.1 | 20 | 21 | 22 | 22.1 |  |
| Baez* | ✓ | NR | ✓ | NR | ? | ? | ? | ? | ? | ? | ? | ? | ? | ? | ? | ? | ? | ? | ? | ? | ? | ? | ? | ? | ? | ? | ? | ? | ? | ? | ? | ? | ? | ? | ? |  |
| Culleton* | ✓ | ✓ | ✓ | NR | ? | ? | ? | ? | ? | ? | ? | ? | ? | ? | ? | ? | ? | ? | ? | ? | ? | ? | ? | ? | ? | ? | ? | ? | ? | ? | ? | ? | ? | ? | ? |  |
| Muntion-Alfaro~ | ✓ | ✓ | ? | NR | ✓ | ✓ | ✓ | ✓ | ✓ | ✓ | NR | NR | ? | ? | ? | ✓ | ✓ | ? | ? | ? | ✓ | ? | ✓ | ? | ? | ✓ | ✓ | NA | ? | ✓ | ? | ✓ | ✓ | NR | NR |  |
| Oikarinen | ✓ | ✓ | NR | NR | ✓ | ✓ | ✓ | ✓ | ✓ | NR | NR | NR | ✓ | ✓ | ✓ | ✓ | NR | NR | ? | ✓ | ? | NR | ✓ | NR | NR | ✓ | ? | NA | ✓ | ✓ | ✓ | ✓ | ✓ | NR | NR |  |
| Schlemmer | ✓ | ✓ | ✓ | NR | ✓ | ✓ | ✓ | ✓ | ✓ | ✓ | NR | NR | ✓ | ✓ | ✓ | ? | ✓ | ✓ | ✓ | NR | ✓ | NR | ? | ? | ✓ | ✓ | ✓ | NA | ✓ | ✓ | ✓ | ✓ | ✓ | ✓ | ✓ |  |
| Tahvonen | ✓ | ✓ | ✓ | NR | ✓ | ✓ | ✓ | ✓ | ? | NR | NR | NR | ✓ | ✓ | ? | ? | NR | ? | ? | ? | NR | NR | NR | NR | NR | ✓ | ? | NA | ? | NR | NR | ✓ | ✓ | NR | NR |  |
| ^x^ See published description of RECORD statement for description of items (Benchimol EI, Smeeth L, Guttmann A, Harron K, Moher D, Petersen I, Sørensen HT, von Elm E, Langan SM, RECORD Working Committee. The REporting of studies Conducted using Observational Routinely-collected health Data (RECORD) statement. PLoS medicine. 2015 Oct 6;12(10):e1001885.)  * Abstract only  ~ Abstract published in English, Full study published in Spanish  ✓ Reported adequately  ? unclear; missing information to make a clear decision, but partial information is reported  NR: not reported  NA: Not applicable | | | | | | | | | | | | | | | | | | | | | | | | | | | | | | | | | | | |  |
